# Up-regulation of METCAM/MUC18 promotes motility, invasion, and tumorigenesis of human breast cancer cells

**DOI:** 10.1186/1471-2407-11-113

**Published:** 2011-03-30

**Authors:** Guo-fang Zeng, Shao-xi Cai, Guang-Jer Wu

**Affiliations:** 1 Bioengineering College, Chongqing University, Chongqing 400044, China; 2 Department of Microbiology and Immunology and the Winship Cancer Institute, Emory University School of Medicine, 1510 Clifton Rd, NE, Room 3022/3027 Rollins Research Center Atlanta, GA, 30322, USA

## Abstract

**Background:**

Conflicting research has identified METCAM/MUC18, an integral membrane cell adhesion molecule (CAM) in the Ig-like gene super-family, as both a tumor promoter and a tumor suppressor in the development of breast cancer. To resolve this, we have re-investigated the role of this CAM in the progression of human breast cancer cells.

**Methods:**

Three breast cancer cell lines were used for the tests: one luminal-like breast cancer cell line, MCF7, which did not express any METCAM/MUC18, and two basal-like breast cancer cell lines, MDA-MB-231 and MDA-MB-468, which expressed moderate levels of the protein.

MCF7 cells were transfected with the human METCAM/MUC18 cDNA to obtain G418-resistant clones which expressed the protein and were used for testing effects of human METCAM/MUC18 expression on *in vitro *motility and invasiveness, and *in vitro *and *in vivo *tumorigenesis. Both MDA-MB-231 and MDA-MB-468 cells already expressed METCAM/MUC18. They were directly used for *in vitro *tests in the presence and absence of an anti-METCAM/MUC18 antibody.

**Results:**

In MCF7 cells, enforced METCAM/MUC18 expression increased *in vitro *motility, invasiveness, anchorage-independent colony formation (*in vitro *tumorigenesis), and *in vivo *tumorigenesis. In both MDA-MB-231 and MDA-MB-468 cells, the anti-METCAM/MUC18 antibody inhibited both motility and invasiveness. Though both MDA-MB-231 and MDA-MB-468 cells established a disorganized growth in 3D basement membrane culture assay, the introduction of the anti-METCAM/MUC18 antibody completely destroyed their growth in the 3D culture.

**Conclusion:**

These findings support the notion that human METCAM/MUC18 expression promotes the progression of human breast cancer cells by increasing their motility, invasiveness and tumorigenesis.

## Background

METCAM (alternative names as MUC18, CD146, S-endo 1, MelCAM, and MCAM), an integral membrane cell adhesion molecule (CAM) in the Ig-like gene super-family, has an immunoglobulin-like extra-cellular domain and a cytoplasmic domain, which contains five consensus sequences potentially phosphorylated by PKA, PKC, and CK2 [1,2]. Thus METCAM/MUC18 is capable of performing the typical functions of CAMs: adhesion (cell-cell and cell-extracellular matrix interactions), response to extra-cellular stimuli, intra-cellular interactions with cytoskeleton, and cross-talk with signaling pathways. In addition, METCAM/MUC18 may regulate tumor dormancy, drive cancer cells to a pre-metastatic niche, and help provide a microenvironment for tumor growth in secondary sites [3-5]. The altered expression of METCAM/MUC18 has been shown to increase cell motility, invasiveness, metastasis, and/or tumorigenesis in a number of cancers, including melanoma and prostate cancer [3,4,6-10]. However, the role of METCAM/MUC18 in the progression of human breast cancer cells has been controversial. Results from two groups appeared to support the notion that METCAM/MUC18 may be a tumor suppressor [11,12]. For example, Shih *et al*. showed in animal studies that over-expression of METCAM/MUC18 suppressed the tumor growth of breast cancer MCF7 cells in SCID mice [11]. In addition, Ouhtit *et al*. [12] recently showed that enforced expression of METCAM/MUC18 in the MDA-MB-231 breast cancer cell line decreased *in vitro *invasiveness. On the other hand, results of two other groups appeared to support the opposite notion that METCAM/MUC18 may play a positive role in the progression of breast cancer [13,14]. Garcia *et al*. showed that increased expression of METCAM/MUC18 was correlated with a poor prognosis in breast carcinoma, suggesting a positive correlation of METCAM/MUC18 expression with breast carcinoma progression [13]. Zabouo *et al*. showed that METCAM/MUC18 is expressed in a subset of epithelial cells in malignant breast cancer and that it may contribute to tumor aggressiveness by promoting malignant cell motility (anti-METCAM/MUC18 antibodies decreased motility of MDA-MB-231 cells and transmigration of the same cells through established human endothelial cell layers and reduced the ability of the cells in healing a wound) [14]. These results are more consistent with the currently well-established positive role of METCAM/MUC18 in the progression of melanoma, prostate cancer, and osteosarcomas [3] and also with the promotion in tumor angiogenesis in tumors [15]. Taken together, more evidence appears to favor the notion that the METCAM/MUC18 expression plays a positive role in the progression of breast cancer cells.

In this report, we reinvestigated the role of METCAM/MUC18 in the progression of breast carcinomas. First, we determined the expression of METCAM/MUC18 in a luminal breast cancer cell line, MCF7, which was used by Shih et al [11]. Furthermore, we also expanded our experiment to include another luminal cell line, SK-BR-3 [16,17]. Similar to Shih et al [11] we transfected MCF7 cells with the human METCAM/MUC18 (huMETCAM/MUC18) cDNA gene and obtained many G418-resistant (G418^R^) clones for testing the effect of enforced expression of the protein on *in vitro *motility, invasiveness, and anchorage-independent colony growth in soft agar. Using the same MCF7 clones, we carried out additional experiments to determine the effects of METCAM/MUC18 expression on *in vivo *tumorigenesis in SCID mice. We also determined the effect of METCAM/MUC18 in a basal cell-like breast cancer cell line, MDA-MB-231, which was used by Ouhtit et al. [12] and Zabouo et al. [14]. Again, we expanded our experiment to include an additional basal cell-like cell line, MDA-MB-468 [16,17]. Furthermore, we tested the effects of an anti-humanMETCAM/MUC18 antibody on *in vitro *motility, invasiveness, and a disorganized growth in a 3D basement membrane culture assay of MDA-MB-231 and MDA-MB-468 cells. From our results, we have presented evidence to support the notion that the METCAM/MUC18 expression plays a positive role in the progression of human breast cancer cells.

## Methods

### Cell lines

Human breast cancer cell lines MCF7, SK-BR-3, MDA-MB-231 and 468 were from ATCC. Media were from Invitrogen/Life Technology/GIBCO/BRL. MCF7 cells were maintained in the EMEM medium containing 10% fetal bovine serum (FBS) (Cellgro/MediaTech) and 10 μg/ml of bovine insulin (Sigma/Aldrich). SK-BR-3 cells were maintained in the McCoy's 5A medium supplemented with 10% FBS. MDA-MB-231 and MDA-MB-468 cells were maintained in the Leibovitz's L-15 medium supplemented with 10% FBS. SK-Mel-28 and DU145 cells from ATCC were maintained in the EMEM medium containing 10 mM of Na-pyruvate and 10% FBS. LNCaP cells from ATCC were maintained in a modified RPMI1640 medium supplemented with 25 mM HEPES buffer, 1 mM Na.pyruvate, 1 mM glutamine, 4.5% glucose and 10% FBS. All G418-resistant (G418^R^) MCF7 clones were grown in the same medium of parental MCF7 cells plus 0.5-1 mg/ml G418 (Cellgro/MediaTech and Hyclone). All cell lines and MCF7 clones/cells were maintained in a humidified 37°C incubator with 5% CO_2 _except that MDA-MB-231 and MDA-MB-468 cell lines were maintained in a humidified 37°C incubator without CO_2_.

### Lipofection of MCF7 cells and selection for METCAM/MUC18-expressing G418^R^-clones

6 × 10^5 ^of MCF7 cells were seeded on 60 mm tissue culture Petri dish plates to give about 50% confluence. After one day of growth, monolayer cells were transfected with a mixture of 30 μg of DEMRIE-C (Invitrogen/Life Technology), or FuGene HD (Roche), and 5 μg of the huMETCAM/MUC18 cDNA gene in the plasmid pcDNA3.1+, which contained a HCMV-IE promoter-driven huMETCAM/MUC18 cDNA gene and a SV40 promoter-driven neomycin-resistant gene. 0.5-1 mg/ml of G418 (active component about 72%) was added to the growth medium after transfection and G418^R^-clones emerged in about two weeks. Clones were transferred and expanded sequentially from 24-well to 12-well, then to 6-well culture plates. Cell lysate of each clone was made by adding100 μl of Western blot lysis buffer to the culture in each well of 6-well plates [18], which were then boiled and kept frozen at -20 C until Western blot analysis [8,9]. The METCAM/MUC18-positive clones in the duplicated 6-well plates were further expanded to T-25 flasks and subsequently to T-75 flasks, then processed and frozen in liquid nitrogen for preservation as stock [8,9]. After 24 colonies were picked, the remaining colonies in the 60-mm plates were trypsinized, mixed and seeded to two T-25 flasks; cells grown in one flask were expanded, made stock, and designated as a pooled clone; cells in another flask were made into Western blot lysate and designated as a pooled clone lysate.

### Cell motility assay

A cell motility assay was carried out according to a published method [19] with minor modifications [8,9]. 2 × 10^5 ^cells of each clone/cell line in 0.4 ml of the growth medium containing 0.1%-BSA were incubated with 7.5 to 15 μg/ml of a chicken anti-huMETCAM/MUC18 antibody [2,10] or the control isotype antibody (chicken IgY) for 30 min and seeded to each top insert of the polycarbonate membrane with a 8.0 μm pore size (Fisher #08-771-12 or Falcon 35-3182) that fits into bottom wells of a companion 12-well plate of the Boyden type Transwell system (Fisher #08-771-22 or Falcon 35-3503). To each bottom-well was added 1.1 ml of the regular growth medium containing 10% FBS. After 6 or 19-20 hours, cells migrating to bottom wells were trypsinized, concentrated by centrifugation, and counted with a haemacytometer. The experiments were repeated three times and means and standard deviations of triplicate values were calculated.

### Cell invasiveness Assay

A cell invasiveness assay was carried out according to a published method [19,20] with minor modifications [8,9]. All procedures were similar to the cell motility assay except before seeding cells to top wells, the porous polycarbonate membrane (with a pore size of 12 μm) at the bottom of each top well was coated with 50 μg of diluted Matrigel (growth factors-reduced and phenol-red free grade, BD Biosciences Cat #354237 or Collaborative Research Cat # 40234C). After 6 hours, cells migrating to the bottom well were processed and counted. Alternatively, the porous polycarbonate membrane (with a pore size of 8 μm) at the bottom of each top well was coated with 150 μg of the diluted Matrigel. After 24 hours, cells migrating to the bottom well were processed and counted. Means and standard deviations of triplicate or six repeated values were calculated.

### Anchorage-independent colony formation in soft agar

The published procedures [21] were followed with slight modifications. 0.95 ml of 0.7% Noble agar was added to each well (a diameter of 2.2 cm and a surface area of 3.8 cm^2^) of a 12-well plate to form an agar plug. 1 × 10^4 ^MCF7 clones/cells in 0.9 ml of medium were mixed with 0.1 ml of 3% Noble agar and seeded onto the agar plug in each well and kept in a humidified 37 C incubator. The number of colonies (20-50 cells per colony) was counted after 14 days.

### 3D basement membrane culture assay

The published procedures of a 3D embedded basement membrane culture assay [22] were followed with slight modifications. Eight-chambered RS glass slides (Fisher Cat #12-565-8, Nunc Lab-TekII chamber slide system, Mfr #154534, 0.2-0.5 ml, 0.7-0.8 cm^2^) were pre-chilled, coated with 30-40 μl of Cultrex (Matrigel prepared from EHS, phenol red-free, growth factor-reduced, Cat #3433-005-01 (5 ml), lot # 17307L8-S, Trevigen) per well to form a thin layer and left to solidify for more than 30 min (and up to 3 hours) at 37°C in a humidified CO_2 _incubator. 0.15 ml of a cell suspension (5.3 × 10^5 ^cells/ml) from healthy MDA-MB-231 or MDA-MB-468 monolayer cultures was aliquot into each 1.5 ml micro-centrifuge tube, to which was added 15 μg/ml of an anti-huMETCAM/MUC18 antibody or the control isotype antibody (chicken IgY), and incubated at room temp for 30 min. The medium was then carefully removed after centrifugation in a pre-cooled Eppendorf Mini Spin micro-centrifuge (115 × g for 4 min) and cells in each tube were re-suspended in 0.075 ml of assay medium, cooled on ice for 15 min, and mixed with an equal volume of chilled 4% Matrigel in assay medium. The final mixture (7.95 × 10^4 ^cells) was added to each chamber already pre-coated with a thin layer of Matrigel and incubated at 37°C for 30 min to 2 hours to allow EHS to gel. 0.2 ml of medium was added on top of the cells-EHS gel and replaced every 2 days. The growth in the 3D basement membrane culture assay was observed daily for 2 to 9 days and photographed with a SPOT digital camera attached to an inverted Nikon microscope.

### Determination of tumorigenesis of MCF7 clones/cells in SCID mice

The guidelines of IACUC were strictly followed for the animal studies. One day before injection hairs surrounding the left second and third nipples of 6-week-old female SCID mice (Charles River) were removed with a Veet hair removal gel cream (nouvelle formula, Reckitt & Benckiser Inc, France). Ten mice were used for the injection of each clone. A single cell suspension from monolayer MCF7 cells of the pooled 2D clone (p18), the 2D-5 clone (p19) or the pooled 3D clone (p18) was prepared, washed with PBS, re-suspended at 5 × 10^6 ^per ml in cold 0.05 ml of EMEM without FBS, mixed with an equal volume of 16 mg/ml of Cultrex, and subcutaneously injected with a gauge #28G1/2 needle under the mammary fat pad of the third left nipple. After injection, the size of tumor was measured with a caliper every week. Tumor volume was calculated by using the formula V = π/6 (d1 × d2)^3/2 ^(mm) ^3 ^[19]. At the endpoint (69 days), mice were euthanatized and tumors from each mouse were excised, fixed in formaldehyde, parafinized, and sectioned for histology and immunohistochemical staining.

### Western blot analysis

Cell and tumor lysates were prepared by addition of a Western blot lysis buffer that contained an anti-proteolysis cocktail, as previously described [8,9]. The protein concentration of each lysate was determined and verified after gel electrophoresis and staining as described [8,9,18]. Western blot analysis was followed as previously described [2,8-10,18,23], except the electro-blotted nitrocellulose membrane was incubated with a primary antibody for 16 hours at 4 C. Our chicken anti-huMETCAM/MUC18 IgY [2] (1/300 dilutions) was used as the primary antibody to detect huMETCAM/MUC18 expression. Primary antibodies for detecting the house-keeping gene products actin and β-tubulin were a goat polyclonal antibody (SC-1615, Santa Cruz Biotech) and a rabbit polyclonal antibody (SC-9104, Santa Cruz Biotech), respectively. The secondary antibodies were rabbit anti-goat (AP106 A, Chemicon) or goat anti-rabbit (AP132A, Chemicon), antibodies. The color was developed by the addition of BCIP/NBT (S3771, Promega). The image of a specific protein band corresponding to METCAM/MUC18 on the membrane was scanned with an Epson Scanner model 1260. The intensity was quantitatively determined by NIH software program Image J version 1.31.

### Histology and immunohistochemistry (IHC) of tumor sections

Paraffin-embedded tissue sections (5 μm) were used. A tissue section of the subcutaneous tumor derived from an LNCaP-expressing clone (LNS239) [3,10] was used as a positive external control for IHC. Tissue sections were de-paraffinized, rehydrated with graded alcohol and PBS, and used for histological staining and IHC, as previously described [9,10].

### Statistical analysis of data

*P *values were obtained from statistical analyses by using the Student's *t *test (one-tailed distribution and type 1) to compare data from experimental groups with the control groups for all the figures except the Wilcox rank sum test was also used for figure nine. Two sets of the data were considered significantly different if the *P *value was < 0.05.

## Results

### METCAM/MUC18 expression in four breast cancer cell lines

We initiated the investigation by comparing the expression of METCAM/MUC18 in four human breast cancer cell lines, MCF7, SK-BR-3, MDA-MB-231, and MDA-MB-468 to cell lines whose levels of METCAM/MUC18 expression are well-characterized: the human melanoma cell line SK-Mel-28, which highly expresses METCAM/MUC18; the prostate cancer cell line DU145, which moderately expresses METCAM/MUC18; and the prostate cancer cell line LNCaP, which does not express METCAM/MUC18.

**Figure 1:**
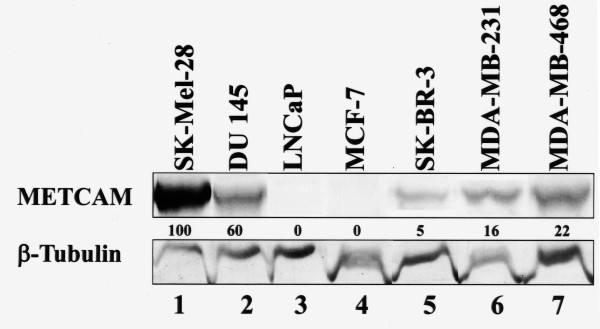
**HuMETCAM/MUC18 expression in four human breast cancer cell lines**. 5 μg proteins of cell lysates were loaded in each lane in Western blot analysis to determine the level of METCAM/MUC18 in various cell lines. Cell lysates from a human melanoma cell line, SK-Mel-18, and a human prostate cancer cell line, DU 145, were used as positive controls (lanes 1-2), and that from a human prostate cancer cell line, LNCaP, as a negative control (lane 3). METCAM/MUC18 expression in cell lysates from four human breast cancer cell lines, MCF7, SK-BR-3, MDA-MB-231, and MDA-MB-468, were determined, as shown in lanes 4 to 7. The number under lane indicates the relative level of METCAMMUC18 of each cell line, assuming that in SK-Mel-28 as 100%. β-tubulin was used as the loading control.

Figure 1 shows that levels of METCAM/MUC18 expression in MCF7, SK-BR-3, MDA-MB-231, and MDA-MB-468 were 0%, 5%, 16%, and 22% of that in SK-Mel-28, respectively. METCAM/MUC18 expression was consistently higher in cell lines established from more malignant breast carcinomas, such as SK-BR-3 and MDA-MB-468, than cell lines from less malignant breast carcinomas, such as MCF7 and MDA-MB-231. We chose MCF7, MDA-MB 231, and MDA-MB-468 for further studies.

### METCAM/MUC18 expression in G418^R^-clones derived from MCF7 cells

**Figure 2:**
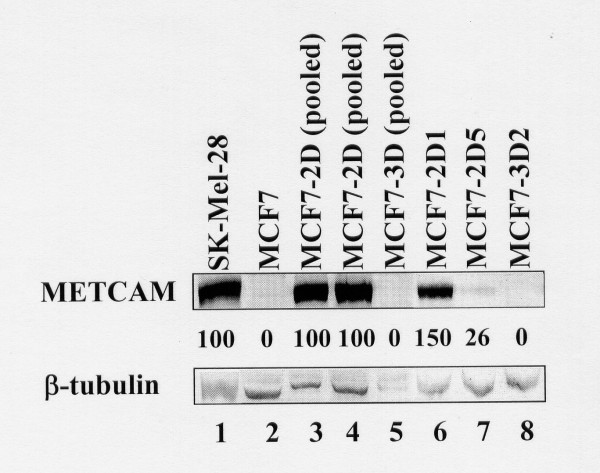
**HuMETCAM/MUC18 expression in various G418^R ^clones of MCF7 cells**. 5 μg proteins of each lysate were loaded in each lane except lane 4, in which 10 μg protein were loaded in Western blot analysis was used to determine the expression of METCAM/MUC18 in various clones/cell lines. Cell lysate from a human melanoma cell line, SK-Mel-18, was used as a positive control (lane 1) and that from the parental human breast cancer cell line MCF7 as a negative control (lane 2). METCAM/MUC18 expressions in cell lysates from five different MCF7 G418^R ^clones (pooled 2D, pooled 3D, 2D1, 2D5, and 3D2) are shown in lanes 3-8. The number under lane indicates the relative level of METCAMMUC18 of each clone, assuming that in SK-Mel-28 as 100%. β-tubulin was used as the loading control.

Since MCF7 did not express any METCAM/MUC18, we transfected METCAM/MUC18 cDNA into MCF7 cells and obtained many G418^R^-clones that expressed different levels of METCAM/MUC18. Figure 2 shows the results of pooled clones and three typical clones when DEMRIE-C (Life Technology) was the transfecting reagent. We found that both DEMRIE-C and FuGene HD (Roche) were excellent transfecting reagents in generating a high percentage of highly expressing clones.

### Effect of METCAM/MUC18 expression on *in vitro *motility and invasiveness of MCF7 cells

**Figure 3:**
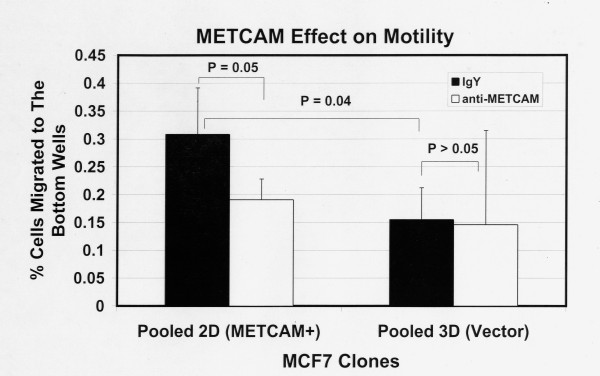
**Effect of huMETCAM/MUC18 expression on *in vitro *motility of MCF7 cells (6 hours)**. The motility of the pooled 2D and 3D clones of MCF7 was determined 6 hours after seeding the cells as described in "Materials and Methods" by using a pore size of 8 μm. 7.5 to 15 μg/ml of the anti-huMCAM/MUC18 antibody (open columns) or the isotype control antibody (chicken IgY) (filled columns) was added to block the motility of these clones. Means and standard deviations of triplicate values of the test are shown.

**Figure 4:**
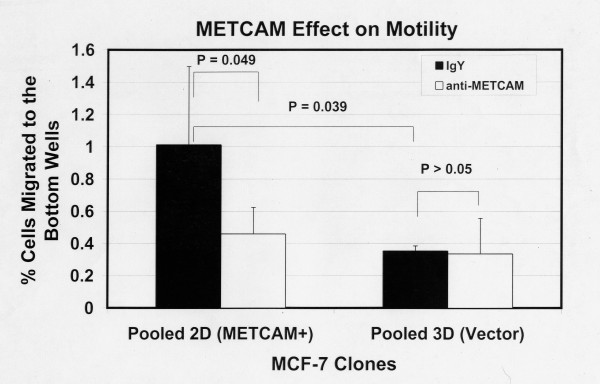
**Effect of huMETCAM/MUC18 expression on *in vitro *motility of MCF7 cells (19 hours)**. The motility of the pooled 2D and 3D clones of MCF7 was determined 19 hours after seeding the cells as described in "Materials and Methods" by using a pore size of 8 μm. 7.5 to 15 μg/ml of the anti-huMCAM/MUC18 antibody (open columns) or the isotype control antibody (chicken IgY) (filled columns) was added to block the motility of these clones. Means and standard deviations of triplicate values of the test are shown.

We used the pooled clones for experiments to eliminate possible effects of individual clonal variations on the results. Figure 3 shows the motility of MCF7 clones 6 hours after seeding the cells and Figure 4 after 19 hours. The results at 6 hours were similar to those at 19 hours, but the data were statistically better at 19 hours. Figure 3 shows that 6 hours after seeding the cells the METCAM/MUC18 expressing clone had a 2-fold higher motility than the vector-control clone. Figure 4 shows that 19 hours after seeding the cells the METCAM/MUC18 expressing clone had a 3-fold higher motility than the vector-control clone. Both Figures 3 and 4 show that the motility of the METCAM/MUC18-expressing clone was significantly reduced in the presence of an anti-huMETCAM/MUC18 antibody, but not the vector clone.

**Figure 5:**
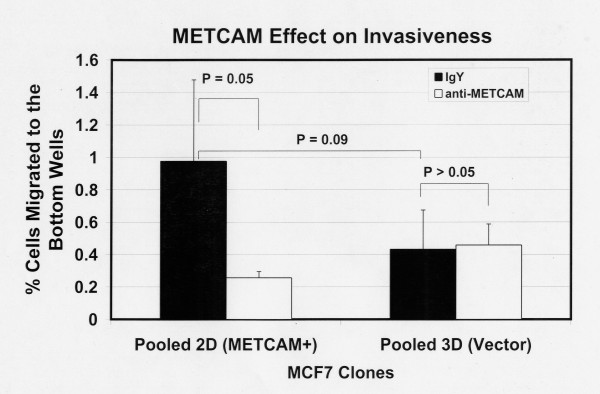
**Effect of huMETCAM/MUC18 expression on *in vitro *invasiveness of MCF7 cells (6 hours)**. The invasiveness of the pooled 2D and 3D clones of MCF7 was determined 6 hours after seeding the cells as described in "Materials and Methods". Since MCF7 clones exhibits a property of very low invasiveness, to observe more cells migrating to the bottom wells 6 hours after seeding in the invasiveness assay the bottom membrane of the top well was coated with 50 μg of Matrigel and a membrane with a pore size of 12 μm used [20]. 7.5 to 15 μg/ml of the anti-huMCAM/MUC18 antibody (open columns) or the isotype control antibody (chicken IgY) (filled columns) was added to block the invasiveness of these clones. Means and standard deviations of triplicate values of the test are shown.

**Figure 6:**
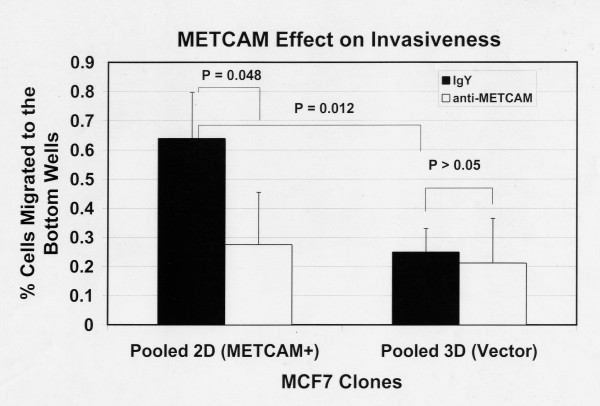
**Effect of huMETCAM/MUC18 expression on *in vitro *invasiveness of MCF7 cells (24 hours)**. The invasiveness of the pooled 2D and 3D clones of MCF7 was determined as 24 hours after seeding the cells described in "Materials and Methods" by coating with 150 μg of Matrigel and using a pore size of 8 μm. 7.5 to 15 μg/ml of the anti-huMCAM/MUC18 antibody (open columns) or the isotype control antibody (chicken IgY) (filled columns) was added to block the invasiveness of these clones. Means and standard deviations of six repeated values of the test are shown.

Figure 5 shows the invasiveness of MCF7 clones 6 hours after seeding the cells and Figure 6 after 24 hours. The results at 6 hours were similar to those at 24 hours, but the data were statistically better at 24 hours. Figure 5 shows that 6 hours after seeding the cells (by coating with 50 μg of Matrigel and using a pore size of 12 μm) the METCAM/MUC18 expressing clone had a somewhat higher invasiveness than the vector-control clone. Figure 6 shows that 24 hours after seeding the cells (by coating with 150 μg of Matrigel and using a pore size of 8 μm) the METCAM/MUC18 expressing clone had a significantly 2.6-fold higher invasiveness than the vector control clone. Both Figures 5 and 6 show that the invasiveness of the METCAM/MUC18-expressing clone was significantly reduced in the presence of an anti-huMETCAM/MUC18 antibody, but not the vector clone.

Taken together, we conclude that METCAM/MUC18 expression increased both the motility and invasiveness of MCF7 cells, and the effect was due to the enforced expression of METCAM/MUC18.

### METCAM/MUC18 expression increased *in vitro *anchorage-independent colony formation of MCF7 cells in soft agar

**Figure 7:**
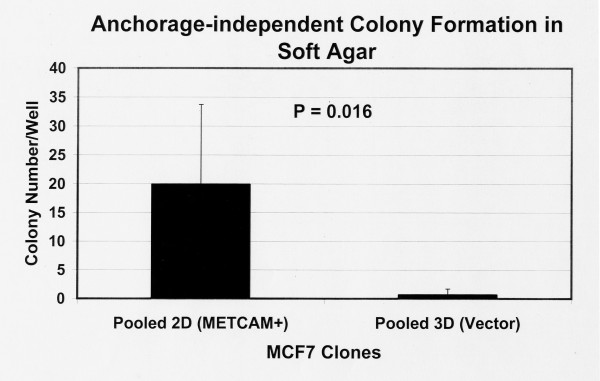
**Effect of huMETCAM/MUC18 expression on MCF7 cells in the formation of anchorage-independent colonies**. Abilities of MCF7 clones to form anchorage-independent colonies were determined by the soft agar colony formation assay as described in "Materials and Methods". Quantitative results of the formation of anchorage-independent colonies by the pooled 2D clone, which was transfected with the huMETCAM/MUC18 cDNA gene, and the pooled 3D clone, which was transfected with the empty vector, are shown. The mean values and standard deviations from the colony numbers in at least four wells are shown.

Anchorage-independent colony formation in soft agar has been successfully used to assess the tumorigenicity of cancer cells *in vitro*, which has been positively correlated to *in vivo *tumorigenicity in animal models [21]. Figure 7 shows quantitative results that the METCAM/MUC18 expressing clone (the pooled 2D clone) had a 10-fold higher ability to form anchorage-independent colonies than the vector-control clone (the pooled 3D clone). We conclude that METCAM/MUC18 expression increased the ability of MCF7 cells in forming anchorage-independent colonies (*in vitro *tumorigenesis).

### Effect of METCAM/MUC18 expression on *in vivo *tumor-take and tumorigenicity of MCF7 clones/cells

In general, MCF7 cells manifest excellent tumorigenicity in athymic nude mice if the cells are co-injected with Matrigel and the mice are supplemented with estrogen pellets [24]. SCID mice were used for the present studies with the aim of repeating the results of Shih et al., who did not implant mice with estrogen pellets [11]. Though we found that tumors were palpable, tumor growth from both the pooled 2D and 3D clones in SCID mice was poor in these immunodeficient mice that were not supplemented with estrogen.

**Figure 8:**
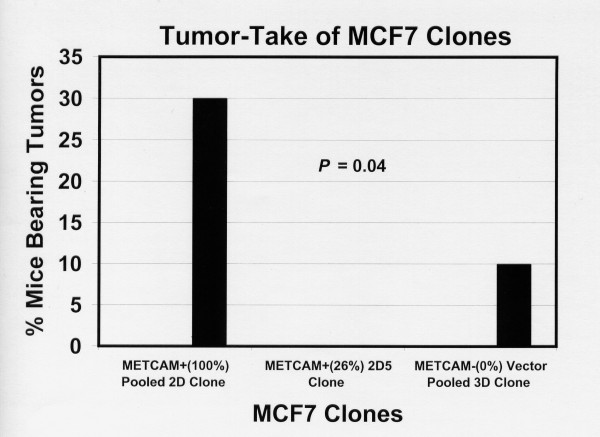
**Effect of huMETCAM/MUC18 expression on the tumor-take of MCF7 cells in SCID mice**. Tumor-take was the number of mice that bore tumor after injection of the cells and was carried out in female SCID mice which were injected with MCF7 cells from the huMETCAM/MUC18-expressing pooled 2D clone and a clone 2D5 and from an empty vector control pooled 3D clone.

**Figure 9:**
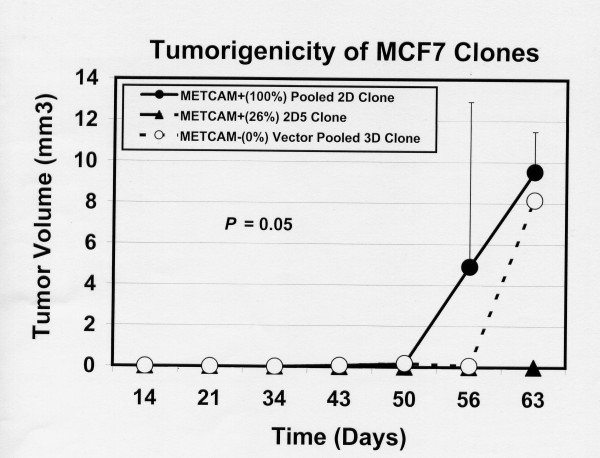
**Effect of huMETCAM/MUC18 expression on the tumorigenicity of MCF7 cells in SCID mice**. Tumorigenicity was determined as described in "Materials and Methods" and is shown as the mean tumor volumes at different time from ten SCID mice for each of the three clones, as described in Figure 8.

Figure 8 shows that the METCAM/MUC18-expressing pooled 2D clone, which expressed high levels of METCAM/MUC18, had a 30% tumor-take. Surprisingly, the METCAM/MUC18-expressing 2D5 clone, which expressed low levels of METCAM/MUC18, did not have any tumor-take. In addition, the vector control clone had a 10% tumor take, which was also unexpected. Figure 9 showed that the tumors of METCAM/MUC18-expressing pooled 2D clone appeared about 6 days earlier than the vector control pooled 3D clone.

The mean final tumor volume of the three tumor-bearing mice injected with the pooled 2D clone was ~4.6 ± 3.1 mm^3^. The final tumor volume of the single tumor-bearing mouse injected with the vector clone was ~14.1 mm^3^.

### Histology and IHC of MCF7 tumors

**Figure 10:**
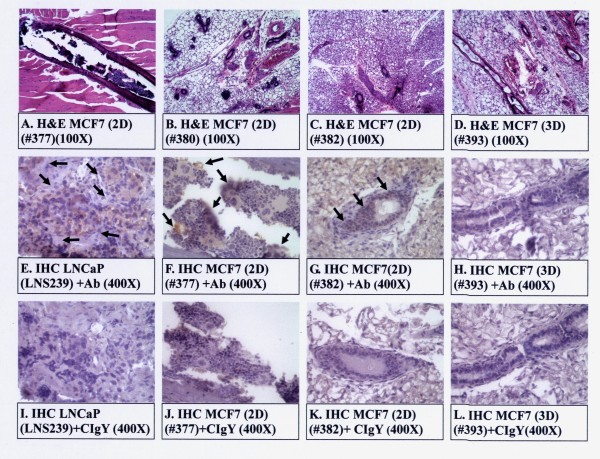
**Histology and immunohistochemistry (IHC) of MCF7 tumors grown in SCID mice**. Histology of tumors derived from pooled 2D and 3D clones are shown in Panels A-D. IHC of tumors from pooled 2D and 3D clones are shown in Panels F-H and J-L. Tumors from the clone LNS239 of LNCaP cells subcutaneously injected to nude mice [3,10,32] was used as a positive control for IHC (panels E & I). The anti-human METCAM/MUC18 antibody (+Ab) was added to IHC in Panels E-H. Arrows show the positively stained cells by the anti-human METCAM/MUC18 antibody in the tumors derived from METCAM/MUC18-expressing MCF7 clones/cells (the pooled 2D clone). The control isotype antibody (CIgY) was added to the IHC in Panels I-L, as negative controls. Tumor sections from the tumors derived from the vector control 3D clone (Panels H&L) were also used as negative controls.

Figure 10 shows the histology of the MCF7 tumors from the pooled 2D and 3D clones (Panels A-D). As shown in Panels A-D (Figure 10), only micro-lesions of tumors were observed, as if the tumors were in dormancy. Figure 10 also shows the IHC of these tumor sections that METCAM/MUC18-specific antigens were expressed in the tumors of the 2D clone (Panels F&G), but not in adjacent tumor sections, in which the control isotype antibody (chicken IgY) was added (Panels J&K). In contrast, METCAM/MUC18-specific antigens were poorly expressed in the tumor of the 3D clone (Panel H), similar to the adjacent tumor section, in which the control isotype antibody (chicken IgY) was added (Panel L). IHC results of the tumor sections were consistent with the *in vitro *METCAM/MUC18 expression levels in both the pooled 2D and 3D clones (shown in Figure 2), suggesting that the tumors were from the injected MCF7 clones/cells.

### Effect of METCAM/MUC18 expression on *in vitro *motility and invasiveness of MDA-MB-231 and MDA-MB-468 cells

**Figure 11:**
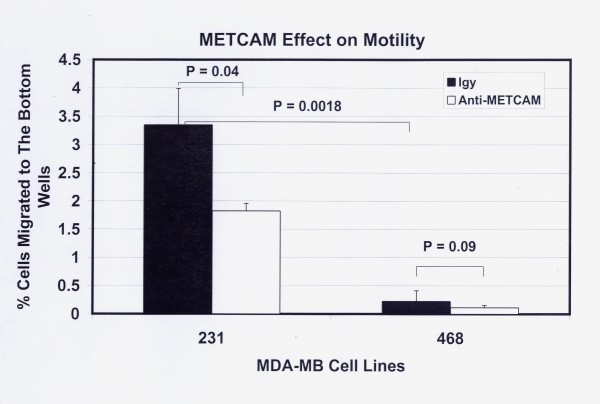
**Effect of huMETCAM/MUC18 expression on *in vitro *motility of MDA-MB-231 and MDA-MB-468 cell lines (6 hours)**. The motility of MDA-MB-231 and MDA-MB-468 cells was determined 6 hours after seeding the cells as described in "Materials and Methods" by using a pore size of 8 μm. 7.5 to 15 μg/ml of the anti-huMCAM/MUC18 antibody (open columns) or the isotype control antibody (chicken IgY) (filled columns) was added to block the motility of these clones. Means and standard deviations of triplicate values of the test are shown.

**Figure 12:**
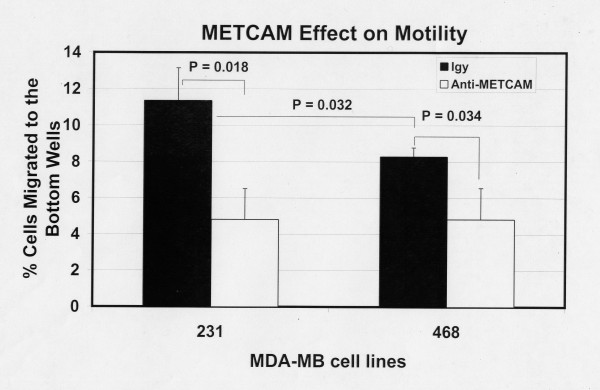
**Effect of huMETCAM/MUC18 expression on *in vitro *motility of MDA-MB-231 and MDA-MB-468 cell lines (20 hours)**. The motility of MDA-MB-231 and MDA-MB-468 cells was determined 20 hours after seeding the cells as described in "Materials and Methods" by using a pore size of 8 μm. 7.5 to 15 μg/ml of the anti-huMCAM/MUC18 antibody (open columns) or the isotype control antibody (chicken IgY) (filled columns) was added to block the motility of these clones. Means and standard deviations of triplicate values of the test are shown.

Figure 11 shows the motility of both MDA-MB-231 and MDA-MB-468 cells 6 hours after seeding the cells and Figure 12 after 20 hours. The results at 6 hours were similar to those at 20 hours, but the data were statistically better at 20 hours. At both time points MDA-MB-231 cells had an 11-fold higher level of motility than the METCAM/MUC18-expressing clones of MCF7 cells (Figures 11 and 12 versus Figures 3 and 4). As also shown in Figures 11 and 12, MDA-MB-231 cells consistently had a significantly higher motility than MDA-MB-468 cells. At 6 hours after seeding the cells MDA-MB-468 cells exhibited motility similar to the METCAM/MUC18-expressing clones of MCF7 cells (Figure 11 versus Figure 3), but at 20 hours after seeding the cells MDA-MB-468 cells had a significant 8-fold higher level of motility than the METCAM/MUC18-expressing clones of MCF7 cells (Figure 12 versus Figure 4). At both time points, MDA-MB-468 had a significantly higher level of motility than the vector control clone of MCF7 cells (Figures 11 and 12 versus Figures 3 and 4). As also shown in Figure 11 and 12, the motility of both these cell lines was significantly reduced in the presence of an anti-huMETCAM/MUC18 antibody.

**Figure 13:**
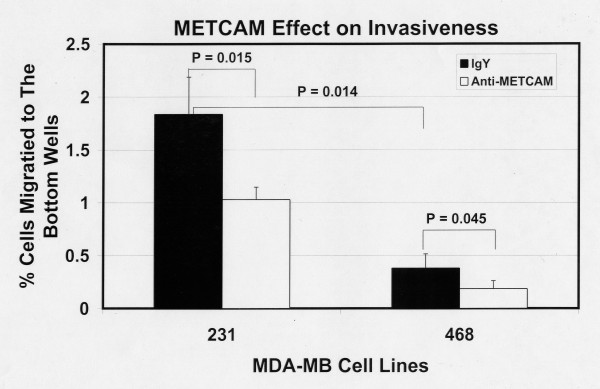
**Effect of huMETCAM/MUC18 expression on *in vitro *invasiveness of MDA-MB-231 and MDA-MB-468 cell lines (6 hours)**. The invasiveness of MDA-MB-231 and MDA-MB-468 cells was determined 6 hours after seeding the cells as described in "Materials and Methods". Similar to Figure 5, in order to observe more cells migrating to the bottom wells 6 hours after seeding in the invasiveness assay the bottom membrane of the top well was coated with 50 μg of Matrigel and a membrane with a pore size of 12 μm used [20]. 7.5 to 15 μg/ml of the anti-huMCAM/MUC18 antibody (open columns) or the isotype control antibody (chicken IgY) (filled columns) was added to block the invasiveness of these clones. Means and standard deviations of triplicate values of the test are shown.

**Figure 14:**
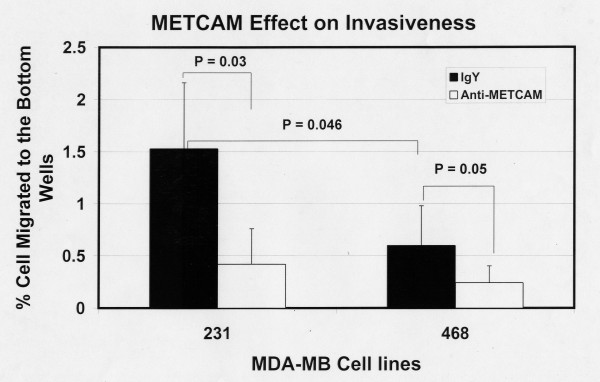
**Effect of huMETCAM/MUC18 expression on *in vitro *invasiveness of MDA-MB-231 and MDA-MB-468 cell lines (24 hours)**. The invasiveness of MDA-MB-231 and MDA-MB-468 cells was determined 24 hours after seeding the cells as described in "Materials and Methods" by coating with 150 μg of Matrigel and using a pore size of 8 μm. 7.5 to 15 μg/ml of the anti-huMCAM/MUC18 antibody (open columns) or the isotype control antibody (chicken IgY) (filled columns) was added to block the invasiveness of these clones. Means and standard deviations of triplicate values of the test are shown.

Figure 13 shows the invasiveness of both MDA-MB-231 and MDA-MB-468 cells 6 hours after seeding the cells and Figure 14 after 24 hours. The results at 6 hours were similar to those at 24 hours, but the data were statistically better at 24 hours. At both time points MDA-MB-231 cells had a 2.5 to 3-fold higher level of invasiveness than the METCAM/MUC18-expressing clones of MCF7 cells (Figures 13 and 14 versus Figures 5 and 6). As also shown in Figures 13 and 14, MDA-MB-231 cells consistently had a higher invasiveness than MDA-MB-468 cells. At 6 hours MDA-MB-468 cells had a lower invasiveness than the METCAM/MUC18-expressing clones of MCF7 cells (Figure 13 versus Figure 5). At 24 hours MDA-MB-468 cells had invasiveness similar to the METCAM/MUC18-expressing clones of MCF7 cells (Figure 14 versus Figure 6). At 6 hours MDA-MB-468 cells had invasiveness similar to the vector control clone of MCF7 (Figure 13 versus Figure 5), but at 24 hours MDA-MB-468 cells had a 2-fold higher level of invasiveness than the vector control clone of MCF7 (Figure 14 versus Figure 6). As also shown in Figures 13 and 14, the invasiveness of both these cell lines was significantly reduced in the presence of an anti-METCAM/MUC18 antibody.

Taken together, we conclude that the endogenous expression of METCAM/MUC18 increased both the motility and invasiveness of MDA-MB-231 and MDA-MB-468 cells.

### Effect of METCAM/MUC18 expression on a 3D basement membrane culture assay of the MDA-MB-231 and MDA-MB-468 cells

**Figure 15:**
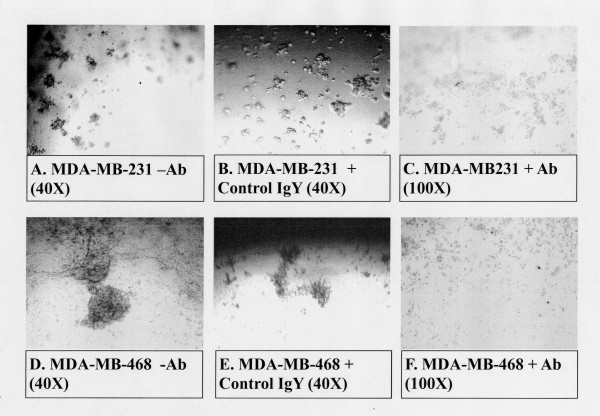
**Effect of huMETCAM/MUC18 expression on a disorganized growth of MDA-MB-231 and MDA-MB-468 cell lines in 3D basement membrane culture assay**. The embedded 3D basement membrane culture assay was carried out as described in "Materials and Methods". Panels A and D, in which no antibody was added, show a disorganized growth of both MDA-MB-231 and MDA-MB-468 cells in 3D culture assay, respectively. Panels B and E, in which the control isotype antibody (Chicken IgY) was added, show results similar to Panels A and D. Panels C and F show that the disorganized growth of both MDA-MB-231 and MDA-MB-468 cells in 3D culture assay was destroyed in the presence of 15 μg/ml of anti-huMETCAM/MUC18 antibody, respectively.

The 3D basement membrane culture has been demonstrated to mimic the *in vivo *growth of normal breast epithelial cells and tumor cells, supporting the former in an organized growth and proper differentiation and the latter in a disorganized growth and manifesting invasiveness [22,25]. Figure 15 shows that both MDA-MB-231 and MDA-MB-468 could establish a disorganized growth in a 3D embedded basement membrane culture (Panels A, B, D and E). Figure 15 also shows that these disorganized growths were destroyed in the presence of an anti-huMETCAM/MUC18 antibody (Panels C and F), suggesting the expression of METCAM/MUC18 is required for them to establish a disorganized growth in a 3D basement membrane culture assay.

## Discussion

We have shown that the expression of METCAM/MUC18 significantly increased the *in vitro *motility, invasiveness, and tumorigenesis of three breast cancer cell lines: MCF7, MDA-MB-231, and MDA-MB-468. The positive effect of METCAM/MUC18 expression on the *in vitro *motility and invasiveness of these clones/cell lines was due to the direct effect of METCAM/MUC18, since these augmented cellular properties of the METCAM/MUC18-expressing clones/cell lines were significantly reduced in the presence of an anti-METCAM/MUC18 antibody. This also was not due to the higher proliferation rate of the METCAM/MUC18-expressing clones/cell lines since we have previously shown that increased expression of METCAM/MUC18 in melanoma and prostate cancer cells did not confer a higher *in vitro *proliferation (growth) rate of these cells and a similar effect was also found in breast cancer cells (data not shown) [8,9]. Furthermore, the *in vitro *doubling time of these cells was longer than the duration of the experiments, especially at the time point of 6 hours. In addition, the cells in the top well were in a serum-free medium, in which they were still alive, but their growth was arrested until they reached to the bottom wells. Taken together, these findings support the notion that METCAM/MUC18 promotes the progression of breast cancer cells. Since MCF7 clones had a lower motility and invasiveness than MDA-MB-231 and MDA-MB-468 cell lines, this may reflect the fact that MCF7 cells exhibit an epithelial morphology, whereas the latter two cell lines a mesenchymal (spindle shape) morphology.

To resolve the contradictory conclusions of the role of METCAM/MUC18 in the progression of breast cancer, we re-investigated effects of METCAM/MUC18 expression on *in vivo *tumorigenesis of MCF7 cells. Using the data from Shih et al. [11] we found that if the tumor volume was calculated using our ellipsoid equation [19], the mean final tumor volume from their vector control clone would have been ~27 ± 7.5 mm^3^, which was about two times larger than our corresponding control clone (14.1 mm^3^), and that from their METCAM-expressing clone would have been ~3.5 ± 2.6 mm^3^, which was similar to our corresponding clone (~4.6 ± 3.1 mm^3^). The results in Shih et al. and this report are both consistent with the results of all previously published work by other groups indicating that without estrogen pellets, the *in vivo *tumorigenesis of MCF7 cells in immuno-deficient mice should be very poor [24], suggesting that in the absence of estrogen the tumor cells may remain in a dormant state. It is unclear why Shih et al. observed a two-fold larger tumor volume from the vector control clone compared to ours. The different results between the two groups may be attributed to the presence of fetal bovine serum, which was the main difference between injection protocols. Fetal bovine serum may partially provide a growth advantage for the vector control clone, but not to the METCAM/MUC18-expressing clone, to proliferate without the need of supplemental estrogen [11].

In addition, Shih et al. found that METCAM/MUC18 expression suppressed the *in vivo *tumorigenesis of MCF7 cells [11]. In contrast, using a more standard method of co-injection of the MCF7 cells with growth-factor reduced Matrigel (without fetal bovine serum) [19,24], we observed a positive effect of METCAM/MUC18 expression on the tumor growth of MCF7 cells in SCID mice. This conclusion was drawn, because the statistical significance of the final single tumor volume from one mouse out of ten from the vector control clone was minimal (though it appeared to be higher than the mean final tumor volume of the three tumors from the METCAM/MUC18 expressing pooled 2D clone). Furthermore, all the mice injected with the cells of the 2D5 clone, which expressed a low level of METCAM/MUC18 and thus was almost like the vector control, did not bear any tumor. Moreover, we found that METCAM/MUC18 expression increased tumor-take and showed a slightly earlier appearance of tumors, though the tumor growth was very poor. We are unable to compare the results of tumor-take from the work of Shih et al., since they did not show this result. Thus, the tumor suppression effect of METCAM/MUC18 in animal studies by Shih et al. [11] could not be reproduced. It is possible that the tumor suppressive effect of METCAM/MUC18 observed by Shih et al. was due to the differential effect of fetal bovine serum.

Another interesting finding (Figure 8) is that the high expression pooled 2D clone had a 30% tumor-take whereas the low expression 2D5 clone had no tumor-take. This suggests that the dosage of METCAM/MUC18 expressed in MCF7 cells may affect *in vivo *tumorigenesis. However, this requires further extensive investigations.

Taken together, we conclude that METCAM/MUC18 plays a positive role in the progression of three breast cancer cell lines, MCF7, MDA-MB-231, and MDA-MB-468. Consistent with this notion, we also observed a similarly positive effect of the METCAM/MUC18 expression on *in vitro *cellular behaviors of another luminal-like cell line, SK-BR-3, and an even more dramatic positive effect of the protein on *in vivo *tumorigenesis of this cell line in nude mice [26,27].

The mechanisms by which huMETCAM/MUC18 expression affects the tumorigenesis of breast cancer cells are poorly understood. Using knowledge derived from tumorigenesis of other tumors [28] and functions of huMETCAM/MUC18 in endothelial cells and melanoma [3,4,15,29-31], huMETCAM/MUC18 expression may increase tumorigenesis by cross-talk with many signaling pathways that affect survival, proliferation, and angiogenesis of tumor cells [3,4]. We therefore predict that enforced expression of huMETCAM/MUC18 may increase tumorigenesis by affecting its key downstream effectors, such as decreasing apoptosis and increasing survival and proliferation of the cells. This notion has been supported by our recent observations that METCAM/MUC18 promotes progression of prostate cancer cells by increasing proliferative indexes (KI67 and PCNA), a survival signaling index (P-AKT/AKT ratio), and pro-angiogenic indexes (VEGF, VEGFR2, and CD31); however, it did not affect apoptosis, which is different from its role in melanoma [32]. Since METCAM/MUC18 promotes both the progression of prostate cancer and breast cancer cells, we anticipate that up-regulation of METCAM/MUC18 may increase expression of similar downstream key parameters during the progression of breast cancer cells. This notion is supported by our recent unpublished results that VEGFR2 levels in METCAM/MUC18-expressing human breast cancer SK-BR-3 tumors were at least two-fold higher than in the vector control SK-BR-3 tumors [26,27].

With regard to the regulation of expression of the METCAM/MUC18 gene in various tumor cells, the METCAM/MUC18 gene is up-regulated at the transcriptional level by PKA via CREB-binding site in the promoter [33] and down-regulated by the transcription factor AP-2 in a human melanoma system [34]. Similar transcriptional regulation of the METCAM/MUC18 gene in breast cancer cells may be used; however, this notion requires future investigation.

## Conclusions

The over-expression of METCAM/MUC18 is likely to promote the tumor progression of luminal-like and basal-like human breast cancer cells. The results of Shih et al. [11], which suggested a tumor-suppressive effect of METCAM/MUC18, were unable to be reproduced using standard methods of animal studies. The positive role of METCAM/MCU18 in the progression of breast cancer is very likely to be similar to that in melanoma and prostate cancer [3,6-10,32]; thus METCAM/MUC18 may be a potential therapeutic target for an alternative treatment of breast cancer [35,36].

## Abbreviations

CAM: cell adhesion molecule; FBS: fetal bovine serum; G418^R^-clones: G418-resistant clone; huMETCAM/MUC18: human METCAM/MUC18; IHC: immunohistochemistry; METCAM: metastasis cell adhesion molecule; PBS: phosphate-buffered saline. SCID mice: severe combined immune deficiency mice.

## Competing interests

The authors declare that they have no competing interests.

## Authors' contributions

GFZ carried out all experiments and analysis of data and prepared the first draft of the manuscript. SXC supported and conceived of the idea for the study of GFZ in USA. GJW conceived of the study, participated in coordination, all experimental designs, problem solving, execution of animal study, and analysis of data, and prepared the final version of the manuscript. All authors read and approved the final manuscript.

## Authors' information

The present work was presented in part at the 101st Annual Meeting of the American Association for Cancer Research in Washington DC, USA, April 17-21, 2010: abstract # 481.

## Pre-publication history

The pre-publication history for this paper can be accessed here:

http://www.biomedcentral.com/1471-2407/11/113/prepub
